# The 'Women's Lifestyle Study', 2-year randomized controlled trial of physical activity counselling in primary health care: rationale and study design

**DOI:** 10.1186/1471-2458-7-166

**Published:** 2007-07-23

**Authors:** Sally B Rose, Beverley A Lawton, C Raina Elley, Anthony C Dowell, Anna J Fenton

**Affiliations:** 1 Women's Health Research Centre, Department of Primary Health Care and General Practice, Wellington School of Medicine and Health Sciences, University of Otago, PO Box 7343, Wellington, New Zealand; 2 Department of General Practice & Primary Health Care, School of Population Health, University of Auckland, Private Bag 92019, Auckland, New Zealand; 3 Department of Primary Health Care and General Practice, Wellington School of Medicine and Health Sciences, University of Otago, PO Box 7343, Wellington, New Zealand; 4 Christchurch Women's Hospital and the Oxford Clinic, 38 Oxford Terrace, Christchurch, New Zealand

## Abstract

**Background:**

Physical inactivity is an independent risk factor for diabetes and heart disease. There is evidence that increasing physical activity can reduce the risk of developing these chronic diseases, but less evidence about effective ways to increase adherence to physical activity. Interventions are therefore needed that produce sustained increases in adherence to physical activity, are cost-effective and improve clinical endpoints.

**Methods:**

The Women's Lifestyle Study is a two year randomized controlled trial involving a nurse-led intervention to increase physical activity in 40–74 year old physically inactive women recruited from primary care. Baseline measures were assessed in a face-to-face interview with a primary care nurse. The intervention involved delivery of a 'Lifestyle script' by a primary care nurse followed by telephone counselling for nine months and a face-to-face nurse visit at six months. Outcome measurements are assessed at 12 and 24 months. The primary outcome is physical activity measured using a validated physical activity questionnaire. Secondary outcomes include blood pressure, weight, waist circumference, physical fitness (step test), serum HbA1c, fasting glucose, lipids, insulin, and quality of life (SF36). Costs were measured prospectively to allow a subsequent cost-effectiveness evaluation if the trial is positive.

**Discussion:**

Due to report in 2008, the Women's Lifestyle Study tests the effectiveness of an enhanced low-cost, evidence-based intervention in increasing physical activity, and improving cardiovascular and diabetes risk indicators over two years. If successful in demonstrating improvements in health outcomes, this randomized controlled trial will be the first to demonstrate long-term cardiovascular and diabetes risk health benefit, in addition to improvements in physical activity, from a sustainable physical activity intervention based in primary care.

**Trial Registration:**

Australian Clinical Trials Registry (ACTR), ACTRN012605000490673.

## Background

Physical inactivity is a major independent risk factor for type 2 diabetes and cardiovascular disease [1]. Evidence suggests 30 minutes of moderate intensity physical activity on most days, or 150 minutes per week is adequate for health benefit [2]. Fewer than 40% of New Zealand adults achieve this amount [3]. The sedentary nature of the population, together with the increasing levels of obesity have contributed to an epidemic of type 2 diabetes in New Zealand and internationally [4]. In New Zealand there are marked disparities in health outcomes for Maori who are the indigenous people of New Zealand (14% of the population) and Pacific people compared with non-Maori [5]. For example, Maori and Pacific people are three times more likely to develop type 2 diabetes than European New Zealanders [6].

Three randomized controlled trials have demonstrated that increasing physical activity to recommended levels and reducing weight can reduce the progression from pre-diabetes to diabetes by approximately 60% [7-9]. However, interventions such as those used in the US Diabetes Prevention Program that involved both dietary and exercise components, are too expensive to be practical from a population perspective. Evaluations of interventions to promote physical activity alone have achieved moderate increases in self-reported physical activity as well as improved cardio-respiratory fitness in sedentary populations [10].

'Exercise on prescription' interventions have been used with some success [11]. For example the 'green prescription' is a primary health care program implemented throughout New Zealand that involves a health professional's verbal and written advice to a patient to be physically active, and three months of telephone support from exercise specialists [12]. A cluster randomized controlled trial demonstrated that the green prescription intervention produced significant improvements in physical activity levels and quality of life amongst 40–79 year old 'less active' adults in primary care over a 12 month period [12]. The proportion of participants that were achieving at least 150 minutes of moderate- or vigorous-intensity leisure activity increased in the intervention group from 18% to 33% over the 12 months [12]. Despite being cost-effective, the incremental increase in adherence to physical activity in those receiving the green prescription was low (10%) compared with the control group, and did not demonstrate significant changes in clinical indices [13,14]. There were trends towards reduced systolic and diastolic blood pressure and total cholesterol levels, which were potentially clinically significant across a population [15]. In fact, few long-term evaluations of physical activity interventions have demonstrated improvements in clinical risk indices corresponding with increases in physical activity. A systematic review of interventions to promote walking found only two studies that achieved increases in both walking and changes in clinical risk factors [16]. One study involved participants with Type 2 diabetes and demonstrated reduced waist measurements [17]. Another study involved participants with established ischemic heart disease and showed improvements in blood pressure control and cholesterol lowering overall [18], but neither study demonstrated differences between intervention and control groups.

Reductions in risk factors for diabetes and cardiovascular disease may be achieved by greater adherence to physical activity. Qualitative analyses have indicated that more follow-up and support may improve adherence over time [19], but little is known about the sustainability of changes, as few trials have followed participants for more than 12 months. Those studies that did found few incremental differences at two years, partly due to the lack of a true control group [20]. The Women's Lifestyle Study was therefore designed using an existing cost-effective intervention, with extended patient support (nine months, instead of three months with a basic green prescription) and the addition of a face-to-face meeting with the nurse prescriber at six months. The trial has a longer follow-up than previous trials (two years), measures changes in clinical endpoints as well as change in physical activity and will assess cost-effectiveness.

## Methods

### Aim

This randomized controlled trial was designed to assess the effectiveness of a primary care physical activity program in increasing adherence to physical activity and reducing diabetes and cardiovascular risk among physically inactive women over a two year period.

### Study population

Inclusion criteria included women aged 40–74 years undertaking less than 150 minutes of at least moderate intensity physical activity per week. Physical activity status was determined by asking a single-item physical activity screening question "As a rule, do you do at least half an hour of moderate or vigorous exercise (such as walking or a sport) on five or more days of the week?" This question has been shown to be highly predictive of low levels of physical activity [21].

Exclusion criteria were based on recommended contraindications to advising physical activity to older adults and included presence of unstable angina, uncontrolled congestive heart failure, unstable arrhythmia or heart valvular disease, progressive or debilitating medical conditions, and severe hypertension (systolic ≥ 200, or diastolic ≥ 120) [22]. The physical activity readiness questionnaire (PAR-Q) was used to assess whether individuals had any medical conditions that might be adversely affected by increasing their physical activity [23]. Individuals with moderate hypertension (average of three recordings systolic ≥ 170, or diastolic ≥ 100), selected heart, joint or balance problems were referred to their family physician for clearance as being safe to undertake moderate intensity physical activity (such as brisk walking) before being randomized at a subsequent visit.

### Recruitment process

Recruitment to the Women's Lifestyle Study took place between November 2004 and November 2005 from 17 primary care practices. Participants were recruited from two sources. The first source was an existing cohort of 50–74 year old women recruited between 1999 and 2002 from 10 primary care practices in Wellington the capital city of New Zealand [24]. The remainder of the participants were 50–70 year old women (40–60 years for Maori and Pacific women) recruited from 13 primary care practices (including 6 practices from which the first group were recruited) in 2004–2005, including two Maori health clinics based on traditional meeting houses (marae) in Wellington. Family physicians at participating practices were asked to identify women in the age group from their practice register excluding patients deemed inappropriate for participation in a physical activity trial. Letters were sent from the practice to those identified as suitable, inviting them to participate in the study. The invitation letter requested that women contact the research team if interested in learning more about the study, using the reply slip and pre-paid envelope supplied. Replies were followed by a phone call from a research nurse to determine eligibility and invite women to an interview. Interviews were held at one of six community healthcare settings.

### Randomisation

Random sequence generation was computer-generated by an independent researcher using STATA 9.1 (StataCorp, College Station, TX, USA) in random blocks sized 2 to 10. Allocation concealment was maintained until after written consent and baseline measures were complete. Sequentially numbered opaque envelopes contained the allocation of treatment groups (intervention or control) and were opened by the nurse after baseline measures were complete.

### Control group

Control participants were told they would have their health followed over the next two years to observe changes over time. They received usual care from their primary care practice.

### Intervention

#### The Lifestyle script

The basic green prescription is prescribed by family physicians and practice nurses and involves the provision of telephone-facilitated activity counselling by community-based Regional Sports Trusts (RST) and their exercise specialists over a three month period [25]. This study used an enhanced green prescription (referred to in this trial as a 'Lifestyle script') that included telephone support from an RST exercise specialist over a nine month period, and involved a face-to-face visit with the primary care nurse at six months to monitor progress and to provide additional support.

Following the completion of baseline measures, participants in the intervention group were given a 'Lifestyle script' that recommended moderate intensity brisk walking (for most participants) or equivalent at a duration and frequency suitable for the individual participant. The script was completed with the participants contact details, as well as clinical details including age, weight, height, waist circumference, smoking status and any relevant medical conditions. The script was faxed to the local RST where a trained exercise specialist with experience in the 'motivational interviewing' counselling technique [26] provided support to participants over the telephone by assisting with choice of activity and the development of an activity plan to fit their lifestyle, goal setting, and ways to overcome personal barriers to physical activity. Ongoing support was provided to participants over a nine month period to help achieve these goals.

#### Six month visit

At six months, intervention participants received a 30 minute face-to-face interview with a primary care research nurse. This meeting was used as an opportunity to establish whether the participant had increased her physical activity to the target level (30 minutes of moderate or vigorous physical activity on five or more days of the week), and was also an opportunity for the nurse to provide encouragement and motivation, and discuss personal health benefits of physical activity. The nurse was provided with a written progress report by the exercise specialist, for use as a discussion tool at the six month visit. Blood pressure, weight and waist were measured and feedback on changes from baseline given to the women. Information on physical activity, tools to assist with choosing appropriate types of activities and motivational aids, such as fridge magnets and activity record charts were also offered.

### Blinding – single blind

Baseline measures were taken prior to allocation of randomisation. Nurses assessing participants at 12 and 24 month follow-up visits were blind to the allocation of treatment group. Participants were asked not to discuss group allocation with the assessing nurse.

### Outcome measures

Study measures were assessed at baseline, 12 and 24 months. The primary outcome measure was physical activity as assessed by the New Zealand physical activity questionnaire long form (NZPAQ-LF) [27]. The NZPAQ-LF is a validated questionnaire that asks participants about physical activity carried out in the past seven days in relation to activity type, context, intensity and duration. Secondary outcomes include: fasting serum HbA1c, lipids, glucose and insulin; weight; waist circumference; blood pressure; physical fitness (measured using a step test) and quality of life (SF36) [28]. Demographic and health data were also collected.

### Data management and quality assurance

Data were entered directly into a customized Microsoft Access database by research nurses at the time of the interview. Daily backups were performed and transferred to the master database at least weekly. Random checks of data entry were performed regularly and corrections made where possible by checking against paper records or in rare cases by phoning participants for confirmation. Clinical recordings outside the normal range were flagged for confirmation of value. All blood samples were tested at the same IANZ accredited laboratory.

### Sample size

On the basis of means, standard deviations and achieved levels of physical activity from previous trials, a sample size of 880 participants was required to detect a minimum of 7% change in the proportion of women reaching the target level of 150 minutes of moderate- or vigorous-intensity physical activity per week, allowing for a 10% attrition rate (α = 0.05, 80% power) [12,29]. This sample size will also be adequate to detect as statistically significant, a difference between the groups in secondary outcomes of 3 mmHg diastolic blood pressure, 0.25 mmol/L serum cholesterol, 0.1 mmol/L HDL and 0.2% HbA1c.

### Statistical methods

Descriptive statistics will be calculated and intervention and control groups checked for balance in demographic, health and outcome measures at baseline. Final analyses will be undertaken using generalized linear mixed models to investigate changes over time in the two groups in the major outcomes. These models allow for repeated measures and can be used for normally distributed, binomial, and ordinal data. An intention-to-treat analysis of all participants enrolled in the study will be carried out according to allocation of randomization, regardless of physical activity adherence. Analyses will commence when all 24 month study measures have been completed so data collection remains blind to any trends in effectiveness until all data is collected.

### Cost-effectiveness analysis

Cost components of the intervention are recorded prospectively from a health-funder and patient perspective. This data will be used in a subsequent cost effectiveness analysis, should the trial prove positive.

## Discussion

Due to report in 2008, the Women's Lifestyle Study tests the effectiveness and sustainability of an enhanced low-cost, evidence-based intervention in increasing physical activity, and improving cardiovascular and diabetes risk indicators over two years. If successful in demonstrating improvements in health outcomes, this randomized controlled trial will be the first to demonstrate long-term cardiovascular and diabetes risk health benefit, in addition to improvements in physical activity, from a sustainable physical activity intervention based in primary care.

Primary health care is an ideal setting to identify adults who are physically inactive and to initiate a brief, cost-effective physical activity intervention. Over 80% of female adults visit their primary care provider (family physician or primary care nurse) each year [30]. Risk factors for diabetes and cardiovascular disease are assessed in primary care and people are used to receiving health-related messages in the context of primary health care. Furthermore, the basic green prescription program is already disseminated throughout New Zealand, delivered to nearly 16,000 patients per year [31]. If the present trial is positive, the data will be assessed to determine whether the enhanced program is cost-effective.

A strength of this study is the inclusion of indigenous Maori, as well as Pacific women who are at greater risk for the development of diabetes in New Zealand. A limitation of the trial is the possibility that participants in the control group are given a basic green prescription as part of their usual care. This potential contamination may dilute the effect of the intervention. However, in a previous trial, the percentage of those in the control group that received a green prescription as part of their usual care was less than 3% so levels of contamination are likely to be low [12]. If successful, this relatively simple intervention delivered through primary care will improve risk factors for cardiovascular disease and diabetes in mid-life and older women, and has the potential to be delivered with success to other sedentary population groups.

## Competing interests

The author(s) declare that they have no competing interests.

## Authors' contributions

All authors contributed to the study design and development of the trial protocol. BL is the principal investigator of the Women's Lifestyle study, and SR the trial manager. SR, CRE, AC and AF are co-investigators. SR and CRE drafted the paper and BL, AC and AF contributed to subsequent drafts. All authors read and approved the final manuscript.

## Funding

Funding was received from the National Heart Foundation of New Zealand (Grant No. 1091 and Grant-in-aid No. 1091 and No. 1222), the Lottery Health Research Grants Board, the Hutt Valley District Health Board, Sport and Recreation New Zealand.

## Ethical approval

This trial was approved by the Central Region Ethics Committee (formerly the Wellington Ethics Committee) in September 2004 (ID number: 04/08/061).

## Trial registration

Australian Clinical Trials Registry (ACTR), ACTRN012605000490673

**Figure 1:**
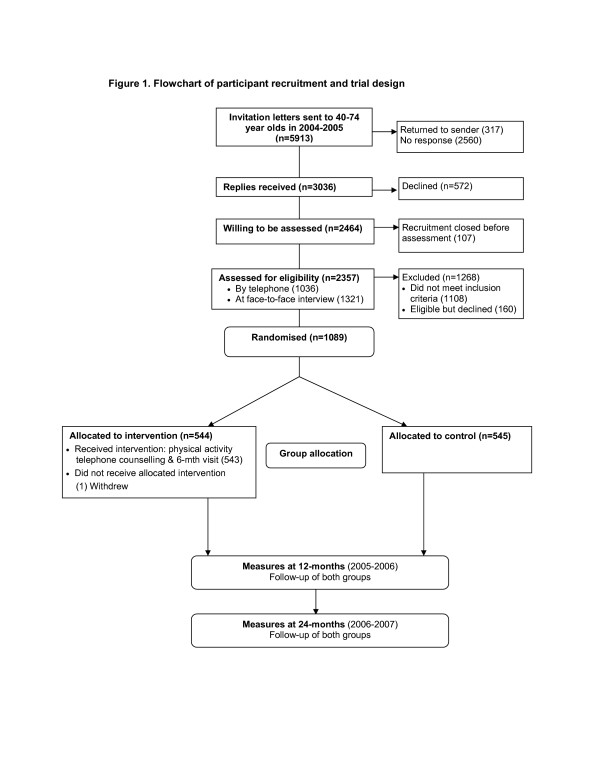
Flowchart of participant recruitment and trial design.

## Pre-publication history

The pre-publication history for this paper can be accessed here:



## Supplementary Material

Additional file 1 CONSORT Checklist of items to include when reporting a randomized trial. Checklist of items included in this randomized controlled trial protocol. Click here for file

## References

[B1] Bassuk SS, Manson JAE (2005). Epidemiological evidence for the role of physical activity in reducing risk of type 2 diabetes and cardiovascular disease. J Appl Physiol.

[B2] Centers for Disease Control and Prevention (1996). Physical activity and health: A report of the Surgeon General..

[B3] van Aalst I, Kazakov D, McLean G (2003). SPARC facts. Results of the New Zealand sport and physical activity surveys (1997-2001).

[B4] PriceWaterhouseCoopers (2001). Type 2 diabetes. Managing for better health outcomes.

[B5] Ajwani S, Blakely T, Robson B, Tobias M, Bonne M (2003). Decades of disparity: Ethnic mortality trends in New Zealand 1980-1999.

[B6] Ministry of Health (2002). Diabetes in New Zealand: Models and Forecasets 1996-2011. Modelling Diabetes: A summary. Public Health Intelligence Occasional Bulletin No 11.

[B7] Diabetes Prevention Program Research Group (2002). Reduction in the incidence of Type 2 Diabetes with lifestyle intervention or Metformin. New Eng J Med.

[B8] Eriksson J JL (1999). Prevention of Type II diabetes in subjects with impaired glucose tolerance: the Diabetes Prevention Study (DPS) in Finland. Study design and 1-year interim report on the feasibility of the lifestyle intervention programme. Diabetologia.

[B9] Pan XR, Li GW, Hu YH, Wang JX, Yang WY, An ZX, Hu ZX, Lin J, Xiao JZ, Cao HB, Liu PA, Jiang XG, Jiang YY, Wang JP, Zheng H, Zhang H, Bennett PH, Howard BV (1997). Effects of diet and exercise in preventing NIDDM in people with impaired glucose tolerance. The Da Qing IGT and Diabetes Study. Diabetes Care.

[B10] Hillsdon M, Foster C, Thorogood M (2005). Interventions for promoting physical activity (review). Cochrane Database of Systematic Reviews.

[B11] Sørensen JB, Skovgaard T, Puggaard L (2006). Exercise on prescription in general practice: A systematic review. Scandinavian Journal of Primary Health Care.

[B12] Elley CR, Kerse N, Arroll B, Robinson E (2003). Effectiveness of counselling patients on physical activity in general practice: cluster randomised controlled trial. BMJ.

[B13] Elley CR, Kerse N, Arroll B, Swinburn B, Ashton T, Robinson E (2004). Cost-effectiveness of physical activity counselling in general practice. N Z Med J.

[B14] Dalziel K, Segal L, Elley CR (2006). Cost utility analysis of physical activity counselling in general practice. Australian & New Zealand Journal of Public Health.

[B15] Cook NR, Cohen J, Hebert PR, Taylor JO, Hennekens CH (1995). Implications of small reductions in diastolic blood pressure for primary prevention. Archives of Internal Medicine.

[B16] Ogilvie D, Foster CE, Rothnie H, Cavill N, Hamilton V, Fitzsimons CF, Mutrie N, on behalf of the Scottish Physical Activity Research Collaboration (2007). Interventions to promote walking: systematic review. BMJ.

[B17] Tudor-Locke C, Bell RC, Myers AM, Harris SB, Ecclestone NA, Lauzon N, Rodger NW (2004). Controlled outcome evaluation of the First Step Program: a daily physical activity intervention for individuals with type II diabetes. International Journal of Obesity & Related Metabolic Disorders: Journal of the International Association for the Study of Obesity.

[B18] Coull AJ, Taylor VH, Elton R, Murdoch PS, Hargreaves AD (2004). A randomised controlled trial of senior Lay Health Mentoring in older people with ischaemic heart disease: The Braveheart Project. Age & Ageing.

[B19] van Aalst I, Daly C (2003). Survey of Green Prescription patients.

[B20] The Writing Group for the Activity Counseling trial Research Group (2001). Effects of Physical activity counseling in primary care: the activity counseling trial: a randomized controlled trial. JAMA.

[B21] Elley CR, Kerse NM, Arroll B (2003). Why target sedentary adults in primary health care? Baseline results from the Waikato Heart, Health, and Activity Study.. Prev Med.

[B22] Huber G, Haskell WL (1997). Medical Clearance for Exercise Program Participation by Older Persons: The Clinical Versus the Public Health Approach.: University of Heidelberg, Germany..

[B23] Canadian Society for Exercise Physiology (2002). Physical Activity Readiness Questionnaire (PAR-Q). http://www.phac-aspc.gc.ca/sth-evs/english/parq.htm.

[B24] Rose S, Lawton B, Dowell T, Fenton AJ (2004). Risk factors for Type 2 diabetes in postmenopausal women: a cross-sectional study. NZ Med J.

[B25] Sport and Recreation New Zealand (1997). The Green Prescription. http://www.sparc.org.nz/getting-active/green-prescription/overview.

[B26] Miller WR (2004). Motivational interviewing in the service of health promotion. Art of Health Promotion in American Journal of Health Promotion.

[B27] McLean G, Tobias M (2004). The New Zealand Physical Activity Questionnaires. Report on the validation and use of the NZPAQ-LF and NZPAQ-SF self-report physical activity survey instruments..

[B28] McHorney CA, Ware JE Jr, Raczek AE (1993). The MOS 36-item short-form health survey (SF-36): II. Psychometric and clinical tests of validity in measuring physical and mental health constructs.. Med Care.

[B29] Stevens W, Hillsdon M, Thorogood M, McArdle D (1998). Cost-effectiveness of a primary care based physical activity intervention in 45-74 year old men and women: a randomised controlled trial.[see comment]. British Journal of Sports Medicine.

[B30] Stevens W, Hillsdon M, Thorogood M, McArdle D (1998). Cost-effectiveness of a primary care based physical activity intervention in 45-74 year old men and women: a randomised controlled trial.. Br J Sports Med.

[B31] O'Neill D (2006). Green Prescription Report to PHARMAC.

